# The Impact of Nursing Resources on Chronic Wound Management: A Cross‐Sectional Analysis

**DOI:** 10.1111/jocn.17804

**Published:** 2025-04-28

**Authors:** Eleanor Turi, Karen B. Lasater, Ariel S. Kamen, Linda H. Aiken, K. Jane Muir

**Affiliations:** ^1^ University of Pennsylvania Perelman School of Medicine National Clinician Scholars Program & Center for Mental Health University of Pennsylvania Leonard Davis Institute of Health Economics Philadelphia Pennsylvania USA; ^2^ University of Pennsylvania School of Nursing Center for Health Outcomes & Policy Research University of Pennsylvania Leonard Davis Institute of Health Economics Philadelphia Pennsylvania USA; ^3^ University of Pennsylvania Perelman School of Medicine Department of Emergency Medicine University of Pennsylvania Leonard Davis Institute of Health Economics Philadelphia Pennsylvania USA

**Keywords:** nursing workforce, patient outcomes, wound care

## Abstract

**Aim:**

Evaluate the relationship between hospital nursing resources and outcomes among patients with chronic wounds.

**Design:**

Cross‐sectional observational.

**Methods:**

Hospital‐level predictors included the nurse work environment, proportion of Bachelor of Science (BSN)‐prepared nurses, and skill mix (i.e., registered nurses [RN] as proportion of nursing personnel). Outcomes included in‐hospital and 30‐day mortality, discharging to a higher level of care and length of stay. Individual‐level nurse data were aggregated to create hospital‐level measures of nursing resources. We utilised multi‐level modelling with nurses nested within hospitals and outcomes at the patient level.

**Data:**

Three datasets from 2021: RN4CAST‐New York/Illinois survey, Medicare Provider Analysis and Review claims and American Hospital Association Annual Survey.

**Results:**

The sample included 34,113 patients with chronic wounds in 215 hospitals in New York and Illinois. In adjusted models, a 1 standard deviation improvement in the work environment was associated with 12% lower odds of in‐hospital mortality, 8% lower odds of discharging to a higher level of care and a shorter length of stay by a factor of 0.96. A 10% increase in BSN composition was associated with 8% reduced odds of in‐hospital mortality and 6% reduced odds of 30‐day mortality. A 10% increase in skill mix was associated with 12% lower odds of in‐hospital mortality and a shorter length of stay by a factor of 0.91.

**Conclusion:**

Improved nursing resources are associated with better outcomes among patients with chronic wounds.

**Implications:**

Nurses manage the care of patients with chronic wounds; thus, hospital investment in nursing resources is imperative for good outcomes.

**Impact:**

Modifiable hospital nursing resources are associated with outcomes among patients with chronic wounds, a complex population.

**Reporting:**

STROBE.

**Summary**

- Identifies modifiable hospital factors linked to improved outcomes.
- Supports investment in nursing resources to enhance care for complex patients.

## 1 Introduction & Background

Chronic wounds—defined as wounds that do not heal in a timely manner—are costly and are associated with significant pain and disability (Armstrong and Meyr, n.d.‐a, n.d.‐b; Nussbaum et al. 2018). Individuals with chronic wounds are often at high risk for adverse outcomes, including the development of infection, chronic pain, amputation and even death (Armstrong et al. 2007; Moxey et al. 2011; Probst et al. 2023; Sen 2023). Chronic wounds pose significant healthcare challenges globally. Africa, Asia and South America are experiencing rapid increases in diabetes and diabetic foot ulcer prevalence, a common chronic wound (Magliano and Boyko 2021; Rigato et al. 2018; Zhang et al. 2017). In Europe, wound care accounts for approximately 2%–4% of total healthcare expenditure, with patients occupying up to 40% of hospital beds and consuming up to half of community healthcare resources (Posnett et al. 2009). Similarly, in Singapore, managing chronic wounds costs around $350 million annually (Graves et al. 2023). In the United States (U.S.), average annual spending per Medicare beneficiary with a chronic wound was $6379 in 2019 (Carter et al. 2023). Chronic wounds affect nearly 10.5 million Medicare beneficiaries (Carter et al. 2023). Clearly, addressing the impact of chronic wounds is critical for improving outcomes and reducing strain on health systems across diverse settings.

Care for patients hospitalised with chronic wounds is nursing intensive and typically involves changing wound dressings multiple times per day, turning patients, assessing wound drainage and ongoing patient education (Kielo et al. 2019). Hospital nurses also manage the complex comorbidities that cause and complicate chronic wounds, which include administering medications, managing communication between multiple provider teams and arranging and providing discharge instructions (Carter et al. 2023; Kielo et al. 2019). As technology advances for chronic wound management (Freedman et al. 2023), nurses remain the frontline clinicians responsible for testing and implementing novel interventions such as new anti‐infective dressings.

While specialised wound care nurses are pivotal in developing standards and providing specialised care for patients with chronic wounds (Bliss et al. 2013; Boyle et al. 2017; Westra et al. 2013), only about one‐third of hospitals in the U.S. have a specialised wound care nurse (Boyle et al. 2017). Patients that do have access often see the specialised nurse for a single consultation, then the care plan is carried out 24/7 by bedside nurses. This disparity in access to specialised wound care may be amplified globally, especially in low and middle‐income countries (Alhassan et al. 2020; Benskin and Benskin 2024; Liu and Wang 2018). Compared to the disparate access to specialised wound care nurses, all hospitalised patients have access to bedside nursing care. Thus, an important question is the extent to which core nursing resources potentially available in general acute hospitals are important for the outcomes of patients with chronic wounds.

A robust literature demonstrates that patient outcomes of all kinds are better when cared for in hospitals where registered nurses are adequately resourced through supportive work environments (i.e., adequate staffing resources, interprofessional collaboration, autonomy, unit decision‐making, foundations for quality care), are qualified at the baccalaureate level (hereafter, Bachelor of Science in Nursing [BSN]), and are present in high proportions relative to other nursing care staff (licensed practical nurses, unlicensed assistive aides) (Aiken et al. 2003, 2014; Bettencourt et al. 2020b; Harrison et al. 2019; Kutney‐Lee et al. 2016; Lasater et al. 2021a, 2024b; Rosenbaum et al. 2024). These characteristics of hospitals—a favourable work environment, higher BSN composition and higher proportion of professional nurses—have been associated with lower odds of patient mortality and readmission, shorter lengths of stay and greater patient satisfaction among patients hospitalised for a range of medical and surgical conditions (Aiken et al. 2003, 2014; Bettencourt et al. 2020b; Harrison et al. 2019; Kutney‐Lee et al. 2016; Lasater et al. 2021a, 2024b; Rosenbaum et al. 2024). The focus of this study is whether these core nursing resources also result in better outcomes for patients with chronic wounds.

## 2 The Study

The objective of this study was to evaluate the relationship between nursing resources available to all hospitalised patients and mortality, discharge to a higher level of care and length of stay among hospitalised patients with chronic wounds. Not all hospitalised patients with chronic wounds have access to specialised wound management teams including advanced practice nurses (Boyle et al. 2017). Thus, this study investigated the extent to which improving nursing resources for all patients would also benefit patients with chronic wounds. It is hypothesised that patients with chronic wounds in hospitals with favourable nurse work environments, more bachelor's‐prepared nurses (higher BSN composition) and more professional nurses as a proportion of all nursing care providers (higher nursing skill mix) will have lower odds of mortality, reduced odds of being discharged to a higher level of care and reduced length of stay.

## 3 Methods

### 3.1 Study Design

This is a cross‐sectional primary data analysis of three linked datasets from 2021: the RN4CAST‐New York/Illinois (NY/IL) survey (Lasater et al. 2021b), Medicare Provider Analysis and Review (MedPAR) patient claims (Research Data Assistance Center (ResDAC), n.d.), and the American Hospital Association (AHA) Annual Survey (American Hospital Association, n.d.). The RN4CAST‐NY/IL survey was approved by the University of Pennsylvania Institutional Review Board (Protocol # 834307). We report our results according to the Strengthening the Reporting of Observational Studies in Epidemiology (STROBE) checklist (available in the Supplemental File).

The RN4CAST‐NY/IL is a survey of nurses as informants of the organisational context where they work (French et al. 2022; Lasater et al. 2021b, 2021c). The RN4CAST‐NY/IL survey data were collected from April to June 2021 via emails sent by the National Council of State Boards of Nursing to all actively licensed RNs in NY and IL. Nonrespondents received follow‐up reminders to complete the survey. Responses were anonymised. The survey took 10–15 min to complete. Nurse respondents provided the name of their employer, which allowed for aggregation of responses from nurses working in the same hospitals. All data were linked through a common hospital identifier. The RN4CAST‐NY/IL data provided the exposure variables of interest: the nurse work environment, BSN composition and nursing skill mix.

MedPAR claims file contains inpatient claims for Medicare fee‐for‐service beneficiaries (Research Data Assistance Center (ResDAC), n.d.). The data provided patient‐level demographics and records of hospitalisations, including admitting diagnosis/procedure, comorbid conditions and outcomes: 30‐day and in‐hospital mortality, source of admission, discharge destination and length of stay. The AHA Annual Survey collects detailed data on U.S. hospitals' organisational structure, service lines, staffing, utilisation metrics and financial performance through an extensive questionnaire completed annually by hospital administrators (American Hospital Association, n.d.). It includes information on over 6000 hospitals nationwide and is widely used in health services research and policy analysis (American Hospital Association, n.d.). The AHA data provided information on hospital bed size, teaching status and technology capabilities.

### 3.2 Sample

Patients were included if they were Medicare beneficiaries (65–99 years old) discharged from one of the study hospitals between 1 January 2021 and 31 December 2021, with a chronic wound primary or secondary diagnosis present on admission. Chronic wounds included pressure ulcers, diabetic ulcers, skin disorders, substance use wounds, venous ulcers, hypertensive ulcers, arterial ulcers and unspecified wounds (see Supplemental File for a detailed list of chronic wound ICD‐10‐CM codes). Patients with burn wounds were excluded because burn wounds require specialised care, often in burn centres with burn care teams (Bettencourt et al. 2020b, 2020a). We also excluded patients who had a length of stay longer than 60 days, which aligns with best practices in nursing health services research (Lasater et al. 2021c, 2021d). Patients with a length of stay longer than 60 days in U.S. general acute care hospitals, the focus of the study, represent long‐term care patients for whom a placement has not been found. Our focus is on patients in general acute care hospitals, not patients who meet criteria for long‐term care. Index hospitalisations were defined as the first admission during the study period for a unique patient. The final patient sample included 34,113 patients with a chronic wound diagnosis present on the index admission.

**FIGURE 1:**
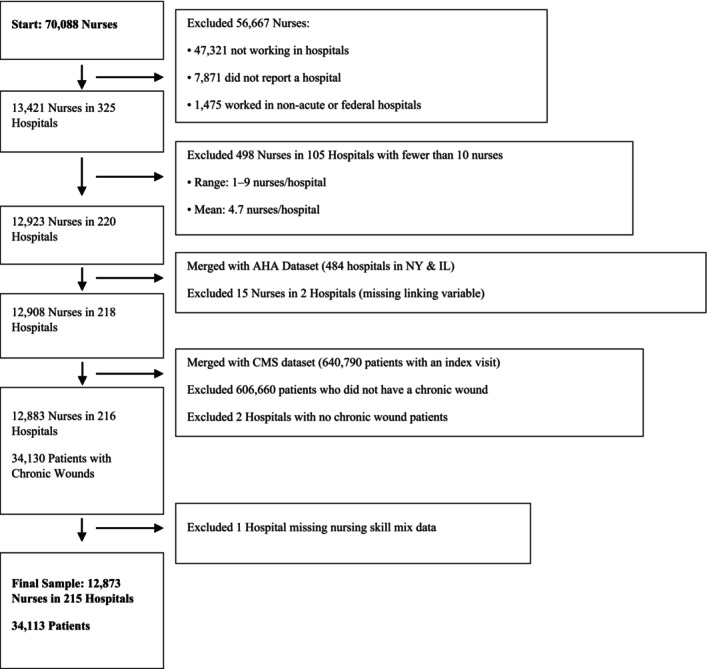
Flow Chart Demonstrating Exclusion of Hospitals, Nurses and Patients due to Missingness.

The study focused on general acute care hospitals, which are hospitals that provide a range of short‐term medical and surgical care. Federal, critical access and psychiatric hospitals were excluded because they are significantly different in characteristics and population served. Federal hospitals are owned and operated by the U.S. government and primarily serve veterans, active‐duty military personnel and Native American communities. Critical access hospitals are designated to improve access to care in underserved areas; they are usually 25 or fewer beds. Psychiatric hospitals provide specialised inpatient care to individuals with severe mental health conditions, such as schizophrenia and bipolar disorder. We excluded 105 hospitals with fewer than 10 nurse respondents per hospital, which is a well‐cited cutoff used in nursing health services research to obtain reliable and representative hospital‐level data (Aiken et al. 2018; Lasater et al. 2024a). There were 4.7 nurses on average in the 105 excluded hospitals. The final hospital sample included 215 hospitals in New York and Illinois. On average, there were 60 nurse survey respondents per hospital (range 10–581 nurse respondents). Figure 1 is a flow chart depicting the selection of our sample.

### 3.3 Measures

#### 3.3.1 Predictors

The main exposures were the nurse work environment, BSN composition and nursing skill mix, all derived from the RN4CAST‐NY/IL survey. The *nurse work environment* was measured using the PES‐5 (Lake et al. 2024), an abbreviated version of the National Quality Forum‐endorsed measure, the Practice Environment Scale of the Nursing Work Index (PES‐NWI) (Lake 2002). The PES‐5 uses one item from each of the five subscales of the PES‐NWI: 1) nurse participation in hospital affairs, (Armstrong and Meyr, n.d.‐a) nursing foundations for quality care, (Armstrong and Meyr, n.d.‐b) nurse manager/leadership ability, (Probst et al. 2023) staffing and resource adequacy and (Moxey et al. 2011) interprofessional collaboration. The PES‐5 has demonstrated good internal reliability with Cronbach's alphas ranging from 0.81 to 0.82 (Lake et al. 2024). Individual responses among nurses working in the same hospital were averaged to create a continuous hospital‐level measure of the nurse work environment. The score was standardised to the mean for interpretation purposes in the regression analyses. Possible scores range from 1 to 4, with a score of 4 indicating the most favourable nurse work environment.

*BSN composition* was measured as the percentage of RNs in each hospital that reported that their highest degree in nursing was a baccalaureate degree or higher (Porat‐Dahlerbruch et al. 2022). In the U.S., a BSN is a degree that prepares nurses for clinical practice, leadership and further education (American Association of Colleges of Nursing 2024). A BSN can be earned through a traditional 4‐year undergraduate programme, an accelerated BSN programme, or an RN‐to‐BSN programme for registered nurses with an associate's degree or diploma (American Association of Colleges of Nursing 2024).

*Nursing skill mix* was defined by the proportion of RNs in the total nursing staff, where total nursing staff is comprised of RNs, licensed practical nurses (LPNs) and unlicensed assistive personnel (UAPs) (Lasater et al. 2024b). Nurses in the RN4CAST‐NY/IL survey reported on the number of RNs, LPNS and UAPs that provided direct care on the unit during their last shift. In the U.S., nurses must complete an accredited diploma, associate's or bachelor's degree and pass the NCLEX‐RN examination to earn an RN licence (National Council of State Boards of Nursing, n.d.).

#### 3.3.2 Patient Outcomes

*In‐hospital mortality* was defined as a death that occurred during the index hospitalisation. *30‐day mortality* was defined as a death that occurred 30 days from the index admission, including deaths that occurred during the hospitalisation and after discharge.

*Discharged to a higher level of care* was defined as whether a patient presented to the hospital from home and was discharged to a skilled nursing facility or inpatient rehabilitation (See Supplemental File for MedPAR data documentation codes used), excluding patients who died in the hospital or within 30 days of the index admission.

*Length of stay* represented the number of days spent in the hospital during the index admission.

#### 3.3.3 Covariates

Patient covariates included age, Elixhauser comorbidities, biological sex and major diagnostic categories (DRG Codes Lookup, n.d.). Hospital covariates included hospital size, teaching status and technology status. Hospital size was categorised as small (≤ 100 beds), medium (101–250 beds) or large (> 250 beds). Teaching status was defined by the proportion of medical residents/fellows to beds and was either nonteaching (no residents/fellows), minor teaching (< 1:4 residents/fellows per bed) or major teaching (≥ 1:4 residents/fellows per bed). High‐technology hospitals were those with capabilities of performing open‐heart surgery or major organ transplantation. Wound care services at the hospital level, measured in the AHA Survey, were highly correlated with hospital teaching status; therefore, we omitted this factor from adjusted models to optimise parsimony.

#### 3.3.4 Data Analysis

Descriptive statistics were conducted, including averages, frequencies and variances to describe the patient and hospital sample. The study included patient (outcomes, patient covariates) and hospital level (exposure variables of nursing resources, hospital characteristics) data. Nurse responses were aggregated to the hospital level, which has previously been verified to produce unbiased and representative estimates of staffing, work environment and educational characteristics of hospitals (Lasater et al. 2019b). To facilitate hospital‐level aggregation of all exposure variables, we identified that there was adequate agreement on all variables across nurses in the hospitals, as confirmed by an intraclass correlation coefficient of > = 0.70. If hospitals had missing data for the exposure variables (nursing resources), they were excluded from the analysis. All correlations of independent variables were less than 0.7 in the correlation matrix and the average variance inflation factor was 3.6, indicating a lack of multicollinearity.

A series of multi‐level logistic regression models were constructed, accounting for clustering of patients and nurses within hospitals. The models constructed evaluated:

1. The unadjusted joint effects of the exposure variables on outcomes and
2. The joint effects of the exposure variables on outcomes, adjusted for nurse and hospital characteristics

For dichotomous variables (i.e., mortality, discharge to a higher level of care), odds ratios (ORs) are reported. For length of stay, a continuous variable with a skewed distribution, incidence rate ratios (IRRs) from a zero‐truncated negative binomial model are reported. All analyses were conducted in STATA 18.0.

## 4 Results

Of the 34,113 Medicare beneficiaries admitted with a chronic wound diagnosis to one of 215 New York or Illinois hospitals, approximately half were male (50.3%) and had an average age of 76.8 years (Table 1). The most common chronic wounds were pressure ulcers (50.0%), diabetic ulcers (18.5%) and unspecified wounds (17.5%). Less common wounds included skin disorders (7.5%), venous ulcers (3.7%), substance use wounds (2.0%) and hypertensive ulcers (0.9%). Patients had an average Elixhauser score of 4.7 (SD = 1.9, range from −17 to +89 with a higher score indicating more complex comorbidities). The majority of patients had hypertension (76.7%) and diabetes (51.3%). The major diagnostic categories are available in the Supplemental File. The most common were infectious & parasitic diseases, systemic or unspecified sites (22.5%), diseases and disorders of the circulatory system (19.3%) and endocrine, nutritional and metabolic diseases and disorders (9.5%). This aligns with the complexity of the patient population and the time period for this study, which overlapped with the COVID‐19 pandemic.

**TABLE 1:** Characteristics and outcomes of 34,113 patients with a chronic wound present on admission.

Characteristic	Number (%)
Age, mean (SD), y	76.8 (7.5)
Sex	
Female	16,960 (49.7)
Male	17,153 (50.3)
Wound type	
Pressure ulcer	17,041 (50.0)
Diabetic ulcer	6320 (18.5)
Unspecified	5968 (17.5)
Skin disorder	2550 (7.5)
Venous ulcer	1248 (3.7)
Substance use wound	680 (2.0)
Hypertensive ulcers	297 (0.9)
Comorbidities	
Hypertension	26,152 (76.7)
Diabetes	17,498 (51.3)
Heart failure	11,914 (34.9)
Moderate or severe renal failure	11,730 (34.4)
COPD	8228 (24.1)
Dementia	7389 (21.7)
Cerebrovascular disease	2929 (8.6)
Drug abuse	671 (2.0)
Elixhauser Score, mean (SD), *n*	4.7 (1.9)
Admitted from	
Self or clinic referral	29,389 (86.2)
Skilled nursing or intermediate care facility	2557 (7.5)
Transfer from another hospital	1673 (4.9)
Other	494 (1.4)
Discharged to	
Home	7040 (20.6)
Home w/home care	8125 (23.8)
Skilled nursing facility	11,905 (34.9)
Inpatient rehab and short‐term hospital	1678 (4.9)
Other	5365 (15.7)
Outcomes	
Discharged to a higher level of care	9721 (28.5)
Length of Stay, mean (SD), days	8.9 (7.9)
Mortality	
In‐hospital	2397 (7.0)
Within 30 days	5057 (14.8)

*Note:* Hypertension and diabetes include complicated and uncomplicated; Other admitted from includes different health care facility, court/law enforcement, unknown, same hospital with a new claim, hospice and ambulatory surgery; Other discharged to includes self‐discharge, another hospital or institution, planned readmissions, hospice or died; Discharged to a higher level of care is defined as coming from home and discharging to a skilled nursing facility or rehabilitation; Mortality is calculated from admission date. Calculations may not add to 100% due to missingness or rounding.

Most patients were admitted from home or clinic referral (86.2%). Over a third of patients were discharged to either a skilled nursing facility (34.9%) or home with home care services (23.8%). Roughly one‐third (28.5%) of patients were discharged to a higher level of care, defined as presenting to the hospital from home or clinic referral and discharging to a skilled nursing facility or inpatient rehabilitation. The average length of stay in the hospital was 8.9 days (SD = 7.9). The in‐hospital mortality rate was 7.0% and the 30‐day mortality rate was 14.8%.

Of the 215 study hospitals, over half were large (> 250 beds, 53%) with high technology capabilities (61%) (Table 2). Approximately 42% were nonteaching hospitals. The mean work environment score was 2.7 (SD = 0.3; based on a range from 1 to 4, where 4 indicates the most favourable work environment) and the average hospital had a BSN composition of 76% (SD = 16%). The nursing skill mix was on average 77% RNs (SD = 8%) to total nursing staff.

**TABLE 2:** Distribution of the hospital and nursing characteristics.

Characteristic	Hospitals (*N* = 215)
Teaching status, no. (%)	
Not	90 (41.8)
Minor	71 (33.0)
Major	54 (25.2)
Bed size, no. (%)	
≤ 100	25 (11.6)
101–250	76 (35.4)
	114 (53.0)
High technology, no. (%)	130 (60.5)
Work Environment, mean (SD)	2.7 (0.3)
BSN composition: Average percentage of nurses in the hospital with the Bachelor's education (SD)	76.4 (16.1)
Nursing skill mix: Average percentage of nursing staff in the hospital with a Registered Nurse licence (SD)	77.2 (8.2)

*Note:* Calculations may not add to 100% due to missingness or rounding.

Abbreviation: SD, standard deviation.

The results of the logistic regression analyses (Models 1 & 2) are presented in Table 3. In models demonstrating the joint effects of nursing resources (Model 1), a one standard deviation increase (improvement) in the nurse work environment score was associated with 15% lower odds of in‐hospital mortality, 6% lower odds of being discharged to a higher level of care, and shorter length of stay by a factor of 0.90. A 10% increase in the hospital BSN composition was associated with 6% lower odds of 30‐day mortality and a longer length of stay by a factor of 1.05. Finally, a 10% increase in hospital nursing skill mix was associated with 15% lower odds of in‐hospital mortality and a shorter length of stay by a factor of 0.89.

**TABLE 3:** Association between nursing resources and patient outcomes.

	Model 1: Joint effects of nursing resources unadjusted (OR, 95% CI)	Model 2: Fully adjusted model with nursing resources (joint) and patient/hospital characteristics (OR, 95% CI)^a^
In‐hospital mortality (Odds ratio)		
Work environment	0.85 (0.78–0.93)**	0.88 (0.82–0.95)**
BSN composition	0.97 (0.92–1.02)	0.92 (0.87–0.98)*
Nursing skill mix	0.85 (0.76–0.94)*	0.88 (0.80–0.97)*
30‐day mortality (Odds ratio)		
Work environment	0.98 (0.94–1.02)	0.98 (0.94–1.03)
BSN composition	0.94 (0.91–0.97)**	0.94 (0.91–0.97)**
Nursing skill mix	0.95 (0.90–1.01)	0.99 (0.94–1.05)
Discharged to a Higher Level of Care (Odds ratio)		
Work environment	0.94 (0.89–0.99)*	0.92 (0.87–0.97)*
BSN composition	1.00 (0.96–1.04)	1.04 (0.99–1.08)
Nursing skill mix	1.02 (0.94–1.11)	1.05 (0.97–1.15)
Length of Stay (Incident rate ratio)		
Work environment	0.90 (0.88–0.93)**	0.94 (0.92–0.97)**
BSN composition	1.05 (1.03–1.07)**	1.00 (0.98–1.03)
Nursing skill mix	0.89 (0.85–0.93)**	0.91 (0.88–0.94)**

*Note:* *p < 0.05; **p < 0.001.

^a^ Models adjusted for nurse work environment, BSN composition, nursing skill mix, patient characteristics, including age, Elixhauser comorbidities, sex and major diagnostic category, and hospital characteristics including teaching and technology status and bed size. Exposures can be interpreted as a 1 SD improvement in the nurse work environment; a 10% increase in the proportion of hospital BSN‐prepared nurses; a 10% increase in the proportion of RNs to total nursing staff.

The fully adjusted models (Model 2) included the joint effects of nursing resources, adjusted for patient and hospital characteristics. All results remained statistically significant but slightly attenuated in Model 2 aside from results pertaining to BSN composition. In the fully adjusted models, the relationship between BSN composition and length of stay was no longer statistically significant. There also was a new statistically significant association between BSN composition and in‐hospital mortality; a 10% increase in the hospital BSN composition was associated with 8% lower odds of in‐hospital mortality.

## 5 Discussion

This study identified an association between improved nursing resources and better outcomes for patients hospitalised with chronic wounds. Findings demonstrate that patients with chronic wounds in hospitals with better nurse work environments, higher BSN composition and a skill mix rich in RNs had lower odds of in‐hospital and 30‐day mortality, being discharged to a higher level of care and shorter lengths of stay. Investing in hospital work environments, BSN composition and nursing skill mix could lead to reductions in length of stay and mortality as well as improved quality of life (through less time in SNFs and more time at home) for patients with chronic wounds.

Despite a large body of research demonstrating the positive impact of specialised wound care nurses on patient outcomes and the impact of nursing resources on other patient populations (Aiken et al. 2003, 2014; Bettencourt et al. 2020b; Bliss et al. 2013; Boyle et al. 2017; Harrison et al. 2019; Kutney‐Lee et al. 2016; Lasater et al. 2021a; Lasater et al. 2024b; Rosenbaum et al. 2024; Westra et al. 2013), no studies have focused on the impact that bedside nursing care may have on health outcomes for patients with chronic wounds. This was the first study to assess how core nursing resources impact outcomes among all hospitalised patients with chronic wounds, including those without access to specialised wound care, which might be the majority of hospitalised patients, especially globally (Boyle et al. 2017; Lindholm and Searle 2016; Posnett and Franks 2008). Our results demonstrate that bedside nursing resources significantly impact outcomes among chronic wound patients, and this may be because resources impact bedside nurses' ability to deliver regular wound care, manage chronic conditions and apply innovations in chronic wound care such as biomaterials and advanced anti‐infective or sealant bandages (Carter et al. 2023; Freedman et al. 2023; Kielo et al. 2019). If nurses experience time constraints due to inadequate staffing or lack of managerial support (i.e., poor nurse work environments), they may be hindered from delivering wound care, chronic disease management and innovative services that may in turn improve care outcomes among patients with chronic wounds.

## 6 Implications for Practice and Research

Further investments in hospital culture are needed to improve nurses' work environments and in turn the delivery of care to patients with chronic wounds. Global surveys of nurses, like this study, consistently find that nurses lack confidence that management in their settings will listen and respond to nurses concerns about care quality (den Breejen‐de Hooge et al. 2021; Muir et al. 2024; Sojane et al. 2016). Including bedside nurses in hospital affairs (e.g., care protocols, pathways for patients with chronic wounds and work policies such as self‐scheduling) may ultimately achieve better outcomes for the same costs through improved outcomes among hospitalised patients with chronic wounds. To improve the nurse work environment, hospitals can turn to evidence‐based organisation change interventions such as Magnet Recognition or Pathway to Excellence (Kutney‐Lee et al. 2015; Lasater et al. 2016; McHugh et al. 2013; Mulvey et al. 2023). Currently, close to 10% of U.S. hospitals have successfully achieved Magnet Recognition (American Nurses Credentialing Center, n.d.; Lasater et al. 2019a). The Magnet model is being widely tested in Europe as well (Aiken et al. 2024; Sermeus et al. 2022). Hospital systems in Europe, North America, Asia and Central America have achieved Pathways to Excellence designation (American Nurses Credentialing Center, n.d.).

While the educational qualifications of nurses are generally thought of as characteristics of individuals, hospital BSN composition is reflective of organisational policies that preferentially favour BSNs in hiring or require newly hired nurses to obtain their BSN within a certain time after being hired (American Association of Colleges of Nursing 2019). These policies can be adopted, strengthened and implemented at the organisation level to ensure favourable patient outcomes (Porat‐Dahlerbruch et al. 2022). The Institute of Medicine 2011 Future of Nursing report recommended the goal of achieving 80% of the U.S. nurse workforce with a BSN by 2020 (Institute of Medicine 2011). Similarly, global efforts have emphasised advancing nursing education as a mechanism to strengthen health systems (Baker et al. 2021). For example, the World Health Organization State of the World's Nursing called for harmonised nursing education standards and the development of global competencies to promote nursing quality and patient safety (Health Workforce (HWF) 2020). Our results demonstrate that hospitals caring for patients with chronic wounds had an average of 76% BSN nurses, indicating room for improvement.

Finally, many hospital executives across the globe are currently considering or implementing team nursing care models (i.e., having one RN oversee the care of lower‐wage nursing staff such as nursing assistants or LPNs) to address shortages of nursing care at the bedside (Lasater et al. 2024b; Prentice et al. 2020; Yang et al. 2023). The findings of this study caution against team nursing models of care and support existing literature demonstrating that having a higher proportion of RNs at the bedside is associated with better patient outcomes and a better return on investment (Lasater et al. 2024b).

This is the first study to comprehensively use MedPAR claims to study the association between nursing resources and outcomes for patients with chronic wounds and is thus an important addition to the field. Future research should test and refine the ICD‐10‐CM coding schema used in this study (see Supplemental File) to comprehensively capture hospitalised patients with chronic wounds in claims data.

## 7 Limitations

This was an observational study, which limits the ability to make causal inferences. The study was conducted in two states, New York and Illinois, with a patient population of elderly Medicare beneficiaries; however, the sample included a wide variety of hospitals that were heterogeneous regarding teaching status, bed size, the nurse work environment, education and nursing skill mix. Patient outcomes were determined through MedPAR claims, which lack clinical detail with respect to wound severity and complications.

## 8 Conclusions

Investments in nursing resources through favourable hospital work environments, higher hospital BSN composition and a nursing skill mix composed of higher proportions of RNs are associated with reduced odds of mortality, lower odds of discharging to a higher level of care and shorter lengths of stay among hospitalised patients with chronic wounds. To advance implementation of clinical pathways and interventions for patients with chronic wounds, hospital leaders should prioritise improving the work environment, BSN composition and nursing skill to ensure high‐quality outcomes for this vulnerable, high‐cost population.

## Author Contributions

E.T. – conceptualisation, formal analysis, investigation, writing – original draft, writing – review and editing; K.B.L. conceptualisation, funding acquisition, supervision, writing – review and editing; A.S.K. – investigation, writing – original draft, writing – review and editing; L.H.A. – conceptualisation, funding acquisition, supervision, writing – review and editing; K.J.M. – conceptualisation, investigation, formal analysis, supervision, writing – original draft, writing – review and editing.

## Disclosure

No patient or public contribution.

## Conflicts of Interest

The authors declare no conflicts of interest.

## Supporting information


Appendix S1.

Appendix S2.

## Data Availability

Research data are not shared.
