# Supplementary material for: The Impact of Nursing Resources on Chronic Wound Management: A Cross‐Sectional Analysis
Source: J Clin Nurs. 2025 Apr 28;35(1):99–108. doi: 10.1111/jocn.17804 (PMC12353443; doi:10.1111/jocn.17804)
Supplement: Supplementary file 1 — Appendix S1. [file JOCN-35-99-s001.docx]

**Supplemental File**

**Supplemental File Table 1:** STROBE Statement—Checklist of items that should be included in reports of cross-sectional studies

|  | Item No | Recommendation | Page number |
| --- | --- | --- | --- |
| **Title and abstract** | 1 | (*a*) Indicate the study’s design with a commonly used term in the title or the abstract | Title page |
|  |  | (*b*) Provide in the abstract an informative and balanced summary of what was done and what was found | 1-2 |
| Introduction | | |  |
| Background/rationale | 2 | Explain the scientific background and rationale for the investigation being reported | 4-5 |
| Objectives | 3 | State specific objectives, including any prespecified hypotheses | 4-5 |
| Methods | | |  |
| Study design | 4 | Present key elements of study design early in the paper | 5 |
| Setting | 5 | Describe the setting, locations, and relevant dates, including periods of recruitment, exposure, follow-up, and data collection | 5-6 |
| Participants | 6 | (*a*) Give the eligibility criteria, and the sources and methods of selection of participants | 6-7 |
| Variables | 7 | Clearly define all outcomes, exposures, predictors, potential confounders, and effect modifiers. Give diagnostic criteria, if applicable | 7-9 |
| Data sources/ measurement | 8* | For each variable of interest, give sources of data and details of methods of assessment (measurement). Describe comparability of assessment methods if there is more than one group | 7-9 |
| Bias | 9 | Describe any efforts to address potential sources of bias | 10 |
| Study size | 10 | Explain how the study size was arrived at | 7-9 & Figure 1 |
| Quantitative variables | 11 | Explain how quantitative variables were handled in the analyses. If applicable, describe which groupings were chosen and why | 7-9 |
| Statistical methods | 12 | (*a*) Describe all statistical methods, including those used to control for confounding | 10 |
|  |  | (*b*) Describe any methods used to examine subgroups and interactions | n/a |
|  |  | (*c*) Explain how missing data were addressed | 10 |
|  |  | (*d*) If applicable, describe analytical methods taking account of sampling strategy | n/a |
|  |  | (*e*) Describe any sensitivity analyses | n/a |
| Results | | |  |
| Participants | 13* | (a) Report numbers of individuals at each stage of study—eg numbers potentially eligible, examined for eligibility, confirmed eligible, included in the study, completing follow-up, and analysed | Figure 1 |
|  |  | (b) Give reasons for non-participation at each stage | n/a |
|  |  | (c) Consider use of a flow diagram | Figure 1 |
| Descriptive data | 14* | (a) Give characteristics of study participants (eg demographic, clinical, social) and information on exposures and potential confounders | 11-12 & Table 1 |
|  |  | (b) Indicate number of participants with missing data for each variable of interest | n/a |
| Outcome data | 15* | Report numbers of outcome events or summary measures | 11 & Table 1 |
| Main results | 16 | (*a*) Give unadjusted estimates and, if applicable, confounder-adjusted estimates and their precision (eg, 95% confidence interval). Make clear which confounders were adjusted for and why they were included | 12 & Table 3 |
|  |  | (*b*) Report category boundaries when continuous variables were categorized | Table 3 |
|  |  | (*c*) If relevant, consider translating estimates of relative risk into absolute risk for a meaningful time period | n/a |
| Other analyses | 17 | Report other analyses done—eg analyses of subgroups and interactions, and sensitivity analyses | n/a |
| Discussion | | |  |
| Key results | 18 | Summarise key results with reference to study objectives | 12-13 |
| Limitations | 19 | Discuss limitations of the study, taking into account sources of potential bias or imprecision. Discuss both direction and magnitude of any potential bias | 15 |
| Interpretation | 20 | Give a cautious overall interpretation of results considering objectives, limitations, multiplicity of analyses, results from similar studies, and other relevant evidence | 12-13 |
| Generalisability | 21 | Discuss the generalisability (external validity) of the study results | 15 |
| Other information | | |  |
| Funding | 22 | Give the source of funding and the role of the funders for the present study and, if applicable, for the original study on which the present article is based | Title page |

*Give information separately for exposed and unexposed groups.

**Note:** An Explanation and Elaboration article discusses each checklist item and gives methodological background and published examples of transparent reporting. The STROBE checklist is best used in conjunction with this article (freely available on the Web sites of PLoS Medicine at http://www.plosmedicine.org/, Annals of Internal Medicine at http://www.annals.org/, and Epidemiology at http://www.epidem.com/). Information on the STROBE Initiative is available at www.strobe-statement.org.

**Supplemental Table 2:** ICD-10 Codes Corresponding to Chronic Wound Type

| **Wound Type** | **ICD-10 Codes** | **Reference** |
| --- | --- | --- |
| Pressure Ulcer | L89* | Carter MJ, DaVanzo J, Haught R, Nusgart M, Cartwright D, & Fife CE. Chronic wound prevalence and the associated cost of treatment in Medicare beneficiaries: changes between 2014 and 2019. |
| Diabetic Ulcer | **Diabetes:** E08* E09* E13* E10* E11*  **AND**  **Chronic Wounds Associated with Diabetes:** L973* L974* L975* | Carter MJ, DaVanzo J, Haught R, Nusgart M, Cartwright D, & Fife CE. Chronic wound prevalence and the associated cost of treatment in Medicare beneficiaries: changes between 2014 and 2019. |
| Venous Ulcer | I830* I832* I8701* I8703* | Carter MJ, DaVanzo J, Haught R, Nusgart M, Cartwright D, & Fife CE. Chronic wound prevalence and the associated cost of treatment in Medicare beneficiaries: changes between 2014 and 2019. |
| Unspecified Wound | L97* L984* **IF NOT** other wound types | Carter MJ, DaVanzo J, Haught R, Nusgart M, Cartwright D, & Fife CE. Chronic wound prevalence and the associated cost of treatment in Medicare beneficiaries: changes between 2014 and 2019. |
| Skin Disorder | E201 E8359 L88 L120 L128 L138 L139 L52 L26 L304 L538 L920 L951 L982 L732 L959 L921 L942 L988 L942 L989 M3303 M3313 M3390 M3393 Q819 Q826 Q282 T66XXXA T3309XA T3409XA T33529A T34529A T33829A T33839A T34829A T3390XA T3399XA T3490XA T3499XA | Carter MJ, DaVanzo J, Haught R, Nusgart M, Cartwright D, & Fife CE. Chronic wound prevalence and the associated cost of treatment in Medicare beneficiaries: changes between 2014 and 2019. |
| Hypertensive Ulcer | I8731* I8733* | Carter MJ, DaVanzo J, Haught R, Nusgart M, Cartwright D, & Fife CE. Chronic wound prevalence and the associated cost of treatment in Medicare beneficiaries: changes between 2014 and 2019. |
| Substance Use Wound | **Substance Use:** F11* T400* T401* T402* T403* T404* T406*  **AND**  **Substance Use Related Wounds:** A480* I96* N02* I800* I801* I8020* I8021* I8022* I8023* I8029* I803 I808 I809 L08 L089 L92 L988 L97* L984* G06* G09* K630* K750* K6812* K6819* L02* K122* L03* M726* M5402* M793* L02* A49* K650* K651* N10* L989* S0120* S01501* S01502* S0180* S21001* S21002* S21101* S21102* S21201* S21202* S31000* S31100* S31102* S31103* S31104* S31105* S41001* S41002* S41101* S41102* S51001* S51002* S51801* S51802* S61001* S61002* S61200* S61201* S61202* S61203* S61204* S61205* S61206* S61207* S61401* S61402* S61501* S61502* S71001* S71002* S71101* S71102* S81001* S81002* S81801* S81802* S91001* S91002* S91101* S91102* S91103* S91104* S91105* S91301* S91302* S58* S68* S98* S88* M87* T80* T14* | Thakrar AP, Lowenstein M, Greysen SR, Delgado MK. Trends in Before Medically Advised Discharges for Patients With Opioid Use Disorder, 2016-2020. *JAMA.* 2023;330(23):2302–2304. doi:10.1001/jama.2023.21288  Hazen A, Pizzicato L, Hom J, Johnson C, Viner KM. Association between discharges against medical advice and readmission in patients treated for drug injection-related skin and soft tissue infections. *J Subst Abuse Treat.* 2021;126:108465. doi:10.1016/j.jsat.2021.108465  Morgan B, Lancaster R, Boyagoda B, Ananda R, Attwood LO, Jacka D, Woolley I. The burden of skin and soft tissue, bone and joint infections in an Australian cohort of people who inject drugs. BMC Infect Dis. 2024 Mar 7;24(1):299. doi: 10.1186/s12879-024-09143-0.  Da Silva D. Xylazine associated wound ICD-10-CM code. Presentation to the CDC in May 2024.  Department of Public Health, City of Philadelphia. Syndromic Surveillance of Infectious Complications of Substance Use. 2021. |
| Arterial Ulcer | **Arterial Disease:** I7025 I70269  **AND**  **Chronic Wounds Associated with Arterial Disease:** L97109 L97209 L97309 L97409 L97509 L97809 L98499 L97509 | Carter MJ, DaVanzo J, Haught R, Nusgart M, Cartwright D, & Fife CE. Chronic wound prevalence and the associated cost of treatment in Medicare beneficiaries: changes between 2014 and 2019. |
| Burns (excluded) | T2* | Carter MJ, DaVanzo J, Haught R, Nusgart M, Cartwright D, & Fife CE. Chronic wound prevalence and the associated cost of treatment in Medicare beneficiaries: changes between 2014 and 2019. |

**Supplemental Table 3:** Leaving to a Higher Level of Care

| **Variable** | **MedPar Data Documentation Code** |
| --- | --- |
| Coming from home or clinic referral | SRC_IP_ADMSN_CD = 5 |
| Leaving to a skilled nursing facility or inpatient rehabilitation | DSCHRG_DSTNTN_CD = 03, 64, 62, 90 |

**Supplemental Table 4:** Descriptive Results of Major Diagnostic Categories for 34,113 Hospitalized Patients with Chronic Wounds

| **Major Diagnostic Category** | **Diagnostic Related Groups** | **Frequency (%)** |
| --- | --- | --- |
| 0 – Pre-MDC | 001-019 | 244 (0.7) |
| 1 – Diseases & disorders of the nervous system | 020-103 | 1,573 (4.61) |
| 2 – Diseases & disorders of the eye | 113-125 | 13 (0.04) |
| 3 - Diseases & disorders of the ear, nose, mouth, and throat | 135-159 | 87 (0.3) |
| 4 - Diseases & disorders of the respiratory system | 163-208 | 3,058 (9.0) |
| 5 - Diseases & disorders of the circulatory system | 212-320 | 6,586 (19.3) |
| 6 - Diseases & disorders of the digestive system | 321-399 | 1,518 (4.5) |
| 7 - Diseases & disorders of the hepatobiliary system & pancreas | 402-446 | 341 (1.0) |
| 8 - Diseases & disorders of the musculoskeletal system & connective tissue | 453-566 | 2,229 (6.5) |
| 9 - Diseases & disorders of the skin, subcutaneous tissue, and breast | 570-607 | 3,219 (9.4) |
| 10 - Endocrine, nutritional, & metabolic diseases & disorders | 614-645 | 3,229 (9.5) |
| 11 - Diseases & disorders of the kidney & urine tract | 650-700 | 2,624 (7.7) |
| 12 - Diseases & disorders of the male reproductive system | 707-730 | 55 (0.2) |
| 13 - Diseases & disorders of the female reproductive system | 734-761 | 66 (0.2) |
| 16 - Diseases & disorders of the blood, blood forming organs, immunologic disorders | 799-816 | 386 (1.1) |
| 17 – Myeloproliferative diseases & disorders, poorly differentiated neoplasms | 820-850 | 141 (0.4) |
| 18 – Infectious & parasitic diseases, systemic or unspecified sites | 853-872 | 7,667 (22.5) |
| 19 – Mental diseases and disorders | 876-887 | 132 (0.4) |
| 20 – Alcohol/drug use & alcohol/drug induced organic mental disorders | 894-897 | 50 (0.2) |
| 21 – Injuries, poisons, & toxic effects of drugs | 901-923 | 232 (0.7) |
| 23 – Factors influencing health status & other contacts with health services | 939-951 | 136 (0.4) |
| 24 – Multiple significant trauma | 955-965 | 63 (0.2) |
| 25 – Human immunodeficiency virus | 969-977 | 28 (0.1) |
| 26 – Other | 981-999 | 436 (1.3) |

**Supplemental Table 5.** Fully Adjusted Model – Outcome of In-Hospital Mortality (Odds Ratio) Adjusted for Patient and Hospital Characteristics

| **Variable** | **Odds Ratio (95% CI)** |
| --- | --- |
| **Main Predictors** | |
| Work Environment | 0.88 (0.82, 0.95)* |
| BSN Preparation | 0.90 (0.85, 0.96)** |
| Skill Mix | 0.88 (0.79, 0.98)* |
| **Demographics** | |
| Age | 1.03 (1.02, 1.03)** |
| Sex | 1.01 (0.92, 1.10) |
| **Elixhauser Comorbidities** | |
| Acquired Immune Deficiency Syndrome (AIDS) | 1.35 (0.68, 2.67) |
| Alcohol Abuse | 0.84 (0.61, 1.16) |
| Deficiency Anemia | 0.72 (0.66, 0.80)** |
| Autoimmune Disease | 0.78 (0.62, 0.99)* |
| Chronic Blood Loss Anemia | 0.66 (0.39, 1.10) |
| Lymphoma Cancer | 1.02 (0.71, 1.46) |
| Leukemia Cancer | 0.83 (0.51, 1.37) |
| Metastatic Cancer | 1.93 (1.62, 2.28)** |
| Carcinoma in Situ (Early-stage cancer) | Omitted (predicts failure perfectly) |
| Solid Tumor Without Metastasis | 1.31 (1.09, 1.57)* |
| Cerebrovascular Disease | 1.14 (0.96, 1.36) |
| Congestive Heart Failure | 1.41 (1.25, 1.58)** |
| Coagulation Deficiencies | 1.78 (1.57, 2.03)** |
| Dementia | 0.90 (0.80, 1.02) |
| Depression | 0.67 (0.57, 0.79)** |
| Uncomplicated Diabetes | 1.10 (0.93, 1.30) |
| Complicated Diabetes | 0.91 (0.82, 1.02) |
| Drug Abuse | 0.81 (0.57, 1.15) |
| Hypertension with Complications | 0.74 (0.65, 0.83)** |
| Uncomplicated Hypertension | 0.66 (0.59, 0.74)** |
| Mild Liver Disease | 0.88 (0.67, 1.15) |
| Severe Liver Disease | 1.22 (0.87, 1.72) |
| Chronic Obstructive Pulmonary Disease | 1.12 (1.01, 1.23)* |
| Neurological Movement Disorders | 0.97 (0.82, 1.15) |
| Other Neurological Disorders | 1.59 (1.41, 1.81)** |
| Epilepsy/Seizure Disorders | 1.00 (0.82, 1.21) |
| Obesity | 0.80 (0.70, 0.91)* |
| Paralysis | 1.05 (0.85, 1.29) |
| Peripheral Vascular Disease | 0.86 (0.75, 0.97)* |
| Psychotic Disorders | 0.73 (0.58, 0.93)* |
| Pulmonary Circulation Disorders | 1.26 (1.08, 1.47)* |
| Moderate Renal (Kidney) Failure | 1.13 (0.98, 1.30) |
| Severe Renal (Kidney) Failure | 1.85 (1.55, 2.20)** |
| Hypothyroidism | 0.85 (0.75, 0.96)* |
| Other Thyroid Disorders | 0.72 (0.52, 1.01) |
| Peptic Ulcer Disease | 0.71 (0.48, 1.05) |
| Valvular Heart Disease | 0.83 (0.71, 0.97)* |
| Weight Loss | 1.51 (1.36, 1.68)** |
| **Major Diagnostic Categories** | |
| Diseases & disorders of the nervous system | 0.94 (0.61, 1.45) |
| Diseases & disorders of the eye | 0.64 (0.24, 1.76) |
| Diseases & disorders of the ear, nose, mouth, and throat | 1.91 (1.23, 2.96)* |
| Diseases & disorders of the respiratory system | 0.54 (0.36, 0.81)* |
| Diseases & disorders of the circulatory system | 0.65 (0.39, 1.07) |
| Diseases & disorders of the digestive system | 1.05 (0.57, 1.92) |
| Diseases & disorders of the hepatobiliary system & pancreas | 0.38 (0.23, 0.63)** |
| Diseases & disorders of the musculoskeletal system & connective tissue | 0.20 (0.11, 0.35)** |
| Diseases & disorders of the skin, subcutaneous tissue, and breast | 0.30 (0.18, 0.49)** |
| Endocrine, nutritional, & metabolic diseases & disorders | 0.37 (0.23, 0.58)** |
| Diseases & disorders of the kidney & urine tract | 0.23 (0.03, 1.70) |
| Diseases & disorders of the male reproductive system | 1.16 (0.46, 2.96) |
| Diseases & disorders of the female reproductive system | 0.33 (0.15, 0.69)* |
| Diseases & disorders of the blood, blood forming organs, immunologic disorders | 1.33 (0.69, 2.57) |
| Myeloproliferative diseases & disorders, poorly differentiated neoplasms | 2.09 (1.38, 3.17)** |
| Infectious & parasitic diseases, systemic or unspecified sites | 0.45 (0.16, 1.25) |
| Mental diseases and disorders | 0.36 (0.06, 2.05) |
| Alcohol/drug use & alcohol/drug induced organic mental disorders | 0.44 (0.18, 1.08) |
| Injuries, poisons, & toxic effects of drugs | 2.60 (1.17, 5.78)* |
| Factors influencing health status & other contacts with health services | 0.95 (0.36, 2.50) |
| Multiple significant trauma | 1.10 (0.28, 4.39) |
| Human immunodeficiency virus | 0.73 (0.41, 1.31) |
| Other | |
| **Hospital Characteristics** |  |
| Minor teaching status (< 1:4 residents/fellows per bed) | 1.21 (1.00, 1.47) |
| Major teaching status (≥ 1:4 residents/fellows per bed) | 1.19 (0.95, 1.50) |
| High technology hospital | 1.02 (0.88, 1.18) |
| Medium hospital bed size (101-250) | 1.37 (1.02, 1.85)* |
| Large hospital bed side (>250) | 1.50 (1.09, 2.06)* |

**Model statistics:**

Wald chi square (69) = 4439.38

Prob > chi2 = 0.0000

Pseudo R2 = 0.1421

**Supplemental Table 6.** Fully Adjusted Model – Outcome of 30-Day Mortality (Odds Ratio) Adjusted for Patient and Hospital Characteristics

| **Variable** | **Odds Ratio (95% Confidence Interval)** |
| --- | --- |
| **Main Predictors** | |
| Work Environment | 0.98 (0.94 - 1.03) |
| BSN Education | 0.91 (0.87 - 0.95)** |
| Skill Mix | 1.01 (0.95 - 1.07) |
| **Demographics** | |
| Age | 1.04 (1.03 - 1.04)** |
| Sex | 0.98 (0.92 - 1.05) |
| **Elixhauser Comorbidities** | |
| Acquired Immune Deficiency Syndrome (AIDS) | 0.85 (0.46 - 1.58) |
| Alcohol Abuse | 0.87 (0.70 - 1.09) |
| Deficiency Anemia | 0.83 (0.77 - 0.89)** |
| Autoimmune Disease | 0.78 (0.66 - 0.94)* |
| Chronic Blood Loss Anemia | 0.75 (0.53 - 1.07) |
| Lymphoma Cancer | 1.17 (0.88 - 1.55) |
| Leukemia Cancer | 1.21 (0.88 - 1.68) |
| Metastatic Cancer | 3.49 (3.05 - 4.00) |
| Carcinoma in Situ (Early-stage cancer) | Omitted (predicts failure perfectly) |
| Solid Tumor Without Metastasis | 1.58 (1.36 - 1.85)** |
| Cerebrovascular Disease | 1.09 (0.97 - 1.24) |
| Congestive Heart Failure | 1.46 (1.34 - 1.59)** |
| Coagulation Deficiencies | 1.54 (1.39 - 1.69)** |
| Dementia | 1.40 (1.28 - 1.53)** |
| Depression | 0.79 (0.70 - 0.88)** |
| Uncomplicated Diabetes | 1.02 (0.90 - 1.15) |
| Complicated Diabetes | 0.84 (0.77 - 0.91)** |
| Drug Abuse | 0.73 (0.53 - 0.99)* |
| Hypertension with Complications | 0.72 (0.65 - 0.79)** |
| Uncomplicated Hypertension | 0.70 (0.64 - 0.76)** |
| Mild Liver Disease | 1.05 (0.86 - 1.26) |
| Severe Liver Disease | 1.57 (1.22 - 2.02)* |
| Chronic Obstructive Pulmonary Disease | 1.14 (1.05 - 1.23)* |
| Neurological Movement Disorders | 1.08 (0.96 - 1.23) |
| Other Neurological Disorders | 1.57 (1.45 - 1.71)** |
| Epilepsy/Seizure Disorders | 1.04 (0.91 - 1.19) |
| Obesity | 0.72 (0.65 - 0.79)** |
| Paralysis | 1.13 (0.99 - 1.29) |
| Peripheral Vascular Disease | 0.89 (0.81 - 0.98)* |
| Psychotic Disorders | 0.85 (0.72 - 1.00)* |
| Pulmonary Circulation Disorders | 1.20 (1.06 - 1.36)* |
| Moderate Renal (Kidney) Failure | 1.13 (1.01 - 1.26)* |
| Severe Renal (Kidney) Failure | 2.04 (1.80 - 2.31)** |
| Hypothyroidism | 0.96 (0.89 - 1.03) |
| Other Thyroid Disorders | 0.61 (0.46 - 0.79)** |
| Peptic Ulcer Disease | 0.84 (0.64 - 1.11) |
| Valvular Heart Disease | 0.91 (0.81 - 1.03) |
| Weight Loss | 2.02 (1.85 - 2.21)** |
| **Major Diagnostic Categories** | |
| Diseases & disorders of the nervous system | 1.97 (1.28 - 3.04)* |
| Diseases & disorders of the eye | 0.89 (0.10 - 7.86) |
| Diseases & disorders of the ear, nose, mouth, and throat | 1.16 (0.55 - 2.48) |
| Diseases & disorders of the respiratory system | 2.66 (1.68 - 4.21)** |
| Diseases & disorders of the circulatory system | 1.01 (0.66 - 1.55) |
| Diseases & disorders of the digestive system | 1.29 (0.79 - 2.09) |
| Diseases & disorders of the hepatobiliary system & pancreas | 2.53 (1.45 - 4.43)* |
| Diseases & disorders of the musculoskeletal system & connective tissue | 0.76 (0.48 - 1.22) |
| Diseases & disorders of the skin, subcutaneous tissue, and breast | 0.60 (0.38 - 0.93)* |
| Endocrine, nutritional, & metabolic diseases & disorders | 0.85 (0.54 - 1.34) |
| Diseases & disorders of the kidney & urine tract | 0.97 (0.61 - 1.53) |
| Diseases & disorders of the male reproductive system | 0.69 (0.24 - 2.05) |
| Diseases & disorders of the female reproductive system | 1.56 (0.72 - 3.38) |
| Diseases & disorders of the blood, blood forming organs, immunologic disorders | 1.01 (0.56 - 1.81) |
| Myeloproliferative diseases & disorders, poorly differentiated neoplasms | 2.64 (1.40 - 4.95)* |
| Infectious & parasitic diseases, systemic or unspecified sites | 2.96 (1.91 - 4.59)** |
| Mental diseases and disorders | 1.21 (0.57 - 2.54) |
| Alcohol/drug use & alcohol/drug induced organic mental disorders | 0.84 (0.27 - 2.55) |
| Injuries, poisons, & toxic effects of drugs | 0.87 (0.45 - 1.67) |
| Factors influencing health status & other contacts with health services | 3.48 (1.71 - 7.09)* |
| Multiple significant trauma | 3.21 (1.55 - 6.67)* |
| Human immunodeficiency virus | 3.12 (1.12 - 8.68)* |
| Other | 1.16 (0.67 - 1.99) |
| **Hospital Characteristics** | |
| Minor teaching status (< 1:4 residents/fellows per bed) | 1.06 (0.94 - 1.18) |
| Major teaching status (≥ 1:4 residents/fellows per bed) | 0.87 (0.77 - 0.99)* |
| High technology hospital | 0.97 (0.88 - 1.08) |
| Medium hospital bed size (101-250) | 1.20 (0.91 - 1.60) |
| Large hospital bed size (>250) | 1.20 (0.90 - 1.61) |

**Model Statistics:**

Wald chi square (70) = 6287.78

Prob > chi2 = 0.0000

Pseudo R2 = 0.1480

**Supplemental Table 7.** Fully Adjusted Model – Outcome of Discharged to a Higher Level of Care (Odds Ratio) Adjusted for Patient and Hospital Characteristics

| **Variable** | **Odds Ratio (95% Confidence Interval)** |
| --- | --- |
| **Main Predictors** | |
| Work Environment | 0.92 (0.87 - 0.97)* |
| BSN preparation | 1.04 (0.99 - 1.08) |
| Skill Mix | 1.05 (0.97 - 1.15) |
| **Demographics** | |
| Age | 1.02 (1.02 - 1.03)** |
| Sex | 1.14 (1.07 - 1.22)** |
| **Elixhauser Comorbidities** | |
| Acquired Immune Deficiency Syndrome (AIDS) | 0.89 (0.60 - 1.30) |
| Alcohol Abuse | 1.69 (1.42 - 2.01)** |
| Deficiency Anemia | 1.08 (1.02 - 1.14)* |
| Autoimmune Disease | 0.88 (0.79 - 0.99)* |
| Chronic Blood Loss Anemia | 1.16 (0.87 - 1.54) |
| Lymphoma Cancer | 0.88 (0.68 - 1.14) |
| Leukemia Cancer | 0.79 (0.58 - 1.07) |
| Metastatic Cancer | 0.94 (0.78 - 1.12) |
| Carcinoma in Situ (Early-stage cancer) | 1.85 (0.63 - 5.43) |
| Solid Tumor Without Metastasis | 0.85 (0.74 - 0.98)* |
| Cerebrovascular Disease | 1.16 (1.05 - 1.28)* |
| Congestive Heart Failure | 1.06 (0.99 - 1.13) |
| Coagulation Deficiencies | 1.03 (0.94 - 1.14) |
| Dementia | 1.01 (0.92 - 1.11) |
| Depression | 1.13 (1.05 - 1.23)* |
| Uncomplicated Diabetes | 0.94 (0.86 - 1.03) |
| Complicated Diabetes | 0.88 (0.84 - 0.93)** |
| Drug Abuse | 0.75 (0.62 - 0.92)* |
| Hypertension with Complications | 1.03 (0.95 - 1.12) |
| Uncomplicated Hypertension | 0.98 (0.92 - 1.05) |
| Mild Liver Disease | 0.95 (0.83 - 1.10) |
| Severe Liver Disease | 1.09 (0.80 - 1.49) |
| Chronic Obstructive Pulmonary Disease | 0.92 (0.86 - 0.98)* |
| Neurological Movement Disorders | 1.04 (0.93 - 1.15) |
| Other Neurological Disorders | 1.37 (1.27 - 1.47)** |
| Epilepsy/Seizure Disorders | 0.92 (0.82 - 1.04) |
| Obesity | 1.23 (1.15 - 1.32)** |
| Paralysis | 0.78 (0.70 - 0.86)** |
| Peripheral Vascular Disease | 1.01 (0.94 - 1.08) |
| Psychotic Disorders | 1.18 (1.04 - 1.34)* |
| Pulmonary Circulation Disorders | 1.08 (0.98 - 1.19) |
| Moderate Renal (Kidney) Failure | 0.99 (0.92 - 1.06) |
| Severe Renal (Kidney) Failure | 1.07 (0.96 - 1.18) |
| Hypothyroidism | 0.97 (0.90 - 1.05) |
| Other Thyroid Disorders | 0.97 (0.81 - 1.17) |
| Peptic Ulcer Disease | 1.26 (1.00 - 1.59) |
| Valvular Heart Disease | 0.92 (0.84 - 1.00)* |
| Weight Loss | 1.38 (1.28 - 1.48)** |
| **Major Diagnostic Categories** | |
| Diseases & disorders of the nervous system | 1.18 (0.90 - 1.55) |
| Diseases & disorders of the eye | 0.60 (0.16 - 2.26) |
| Diseases & disorders of the ear, nose, mouth, and throat | 0.35 (0.18 - 0.71)* |
| Diseases & disorders of the respiratory system | 0.68 (0.51 - 0.90)* |
| Diseases & disorders of the circulatory system | 0.68 (0.51 - 0.89)* |
| Diseases & disorders of the digestive system | 0.54 (0.41 - 0.72)** |
| Diseases & disorders of the hepatobiliary system & pancreas | 0.51 (0.34 - 0.77)* |
| Diseases & disorders of the musculoskeletal system & connective tissue | 1.49 (1.14 - 1.96)* |
| Diseases & disorders of the skin, subcutaneous tissue, and breast | 0.61 (0.46 - 0.81)** |
| Endocrine, nutritional, & metabolic diseases & disorders | 0.62 (0.47 - 0.81)** |
| Diseases & disorders of the kidney & urine tract | 0.80 (0.61 - 1.04) |
| Diseases & disorders of the male reproductive system | 1.10 (0.61 - 1.98) |
| Diseases & disorders of the female reproductive system | 0.38 (0.21 - 0.69)* |
| Diseases & disorders of the blood, blood forming organs, immunologic disorders | 0.53 (0.38 - 0.74)** |
| Myeloproliferative diseases & disorders, poorly differentiated neoplasms | 0.36 (0.21 - 0.62)** |
| Infectious & parasitic diseases, systemic or unspecified sites | 0.91 (0.70 - 1.20) |
| Mental diseases and disorders | 1.34 (0.85 - 2.12) |
| Alcohol/drug use & alcohol/drug induced organic mental disorders | 0.76 (0.39 - 1.45) |
| Injuries, poisons, & toxic effects of drugs | 0.50 (0.34 - 0.73)** |
| Factors influencing health status & other contacts with health services | 1.14 (0.67 - 1.93) |
| Multiple significant trauma | 2.82 (1.33 - 5.99)* |
| Human immunodeficiency virus | 1.71 (0.75 - 3.87) |
| Other | 0.80 (0.57 - 1.11) |
| **Hospital Characteristics** | |
| Minor teaching status (< 1:4 residents/fellows per bed) | 0.95 (0.82 - 1.10) |
| Major teaching status (≥ 1:4 residents/fellows per bed) | 0.84 (0.72 - 0.98)* |
| High technology hospital | 0.86 (0.75 - 0.99)* |
| Medium hospital bed size (101-250) | 1.17 (0.95 - 1.44) |
| Large hospital bed size (>250) | 1.17 (0.95 - 1.46) |

**Model Statistics:**

Wald chi square (71) = 2069.73

Prob > chi2 = 0.0000

Pseudo r2 = 0.0351

**Supplemental Table 8.** Fully Adjusted Model – Outcome of Length of Stay (Incident Rate Ratio) Adjusted for Patient and Hospital Characteristics

| **Variable** | **Incidence Rate Ratio (95% Confidence Interval)** |
| --- | --- |
| **Main Predictors** | |
| Work Environment | 0.95 (0.92, 0.97)** |
| BSN Preparation | 0.99 (0.97, 1.01) |
| Skill Mix | 0.91 (0.88, 0.94)** |
| **Demographics** | |
| Age | 0.99 (0.99, 0.99)** |
| Sex | 0.99 (0.97, 1.01) |
| **Elixhauser Comorbidities** | |
| Acquired Immune Deficiency Syndrome (AIDS) | 0.94 (0.82, 1.08) |
| Alcohol Abuse | 1.07 (1.02, 1.13)* |
| Deficiency Anemia | 1.06 (1.04, 1.09)** |
| Autoimmune Disease | 0.95 (0.91, 0.99)* |
| Chronic Blood Loss Anemia | 1.14 (1.05, 1.23)* |
| Lymphoma Cancer | 1.03 (0.95, 1.12) |
| Leukemia Cancer | 1.14 (1.03, 1.25)* |
| Metastatic Cancer | 1.05 (0.99, 1.11) |
| Carcinoma in Situ (Early-stage cancer) | 1.99 (1.37, 2.90)** |
| Solid Tumor Without Metastasis | 1.03 (0.98, 1.09) |
| Cerebrovascular Disease | 0.98 (0.94, 1.02) |
| Congestive Heart Failure | 1.09 (1.06, 1.12)** |
| Coagulation Deficiencies | 1.09 (1.05, 1.13)** |
| Dementia | 1.05 (1.02, 1.08)** |
| Depression | 0.97 (0.94, 1.00) |
| Uncomplicated Diabetes | 0.93 (0.90, 0.97)** |
| Complicated Diabetes | 1.05 (1.03, 1.08)** |
| Drug Abuse | 1.00 (0.93, 1.07) |
| Hypertension with Complications | 0.99 (0.96, 1.02) |
| Uncomplicated Hypertension | 0.90 (0.87, 0.93)** |
| Mild Liver Disease | 1.02 (0.97, 1.07) |
| Severe Liver Disease | 1.10 (1.01, 1.19)* |
| Chronic Obstructive Pulmonary Disease | 0.96 (0.95, 0.98)** |
| Neurological Movement Disorders | 0.98 (0.95, 1.02) |
| Other Neurological Disorders | 1.16 (1.14, 1.19)** |
| Epilepsy/Seizure Disorders | 1.03 (0.98, 1.08) |
| Obesity | 1.06 (1.03, 1.08)** |
| Paralysis | 1.13 (1.10, 1.17)** |
| Peripheral Vascular Disease | 1.10 (1.07, 1.13)** |
| Psychotic Disorders | 1.02 (0.97, 1.06) |
| Pulmonary Circulation Disorders | 1.12 (1.07, 1.16)** |
| Moderate Renal (Kidney) Failure | 0.97 (0.94, 1.00)* |
| Severe Renal (Kidney) Failure | 1.11 (1.07, 1.15)** |
| Hypothyroidism | 0.94 (0.91, 0.96)** |
| Other Thyroid Disorders | 0.96 (0.90, 1.03) |
| Peptic Ulcer Disease | 1.37 (1.28, 1.48)** |
| Valvular Heart Disease | 1.00 (0.97, 1.03) |
| Weight Loss | 1.31 (1.28, 1.35)** |
| **Major Diagnostic Categories** | |
| Diseases & disorders of the nervous system | 0.28 (0.25, 0.30)** |
| Diseases & disorders of the eye | 0.28 (0.16, 0.50)** |
| Diseases & disorders of the ear, nose, mouth, and throat | 0.24 (0.18, 0.32)** |
| Diseases & disorders of the respiratory system | 0.35 (0.33, 0.38)** |
| Diseases & disorders of the circulatory system | 0.28 (0.26, 0.29)** |
| Diseases & disorders of the digestive system | 0.27 (0.25, 0.29)** |
| Diseases & disorders of the hepatobiliary system & pancreas | 0.27 (0.24, 0.30)** |
| Diseases & disorders of the musculoskeletal system & connective tissue | 0.29 (0.27, 0.32)** |
| Diseases & disorders of the skin, subcutaneous tissue, and breast | 0.23 (0.21, 0.24)** |
| Endocrine, nutritional, & metabolic diseases & disorders | 0.26 (0.24, 0.28)** |
| Diseases & disorders of the kidney & urine tract | 0.24 (0.23, 0.26)** |
| Diseases & disorders of the male reproductive system | 0.25 (0.20, 0.31)** |
| Diseases & disorders of the female reproductive system | 0.25 (0.21, 0.29)** |
| Diseases & disorders of the blood, blood forming organs, immunologic disorders | 0.22 (0.19, 0.25)** |
| Myeloproliferative diseases & disorders, poorly differentiated neoplasms | 0.37 (0.30, 0.45)** |
| Infectious & parasitic diseases, systemic or unspecified sites | 0.34 (0.32, 0.37)** |
| Mental diseases and disorders | 0.30 (0.26, 0.35)** |
| Alcohol/drug use & alcohol/drug induced organic mental disorders | 0.30 (0.22, 0.40)** |
| Injuries, poisons, & toxic effects of drugs | 0.25 (0.21, 0.29)** |
| Factors influencing health status & other contacts with health services | 0.16 (0.13, 0.20)** |
| Multiple significant trauma | 0.32 (0.26, 0.39)** |
| Human immunodeficiency virus | 0.46 (0.33, 0.63)** |
| Other | 0.42 (0.38, 0.47)** |
| **Hospital Characteristics** | |
| Minor teaching status (< 1:4 residents/fellows per bed) | 1.08 (1.01, 1.16)* |
| Major teaching status (≥ 1:4 residents/fellows per bed) | 1.18 (1.10, 1.28)** |
| High technology hospital | 0.95 (0.90, 1.00) |
| Medium hospital bed size (101-250) | 1.15 (1.01, 1.30)* |
| Large hospital bed size (>250) | 1.27 (1.12, 1.45)** |

**Model Statistics:**

Wald chi square (71) = 9622.82

Prob > chi2 = 0.0000

Pseudo R2 = 0.0289
